# A208 ENDOSCOPIC SUBMUCOSAL DISSECTION (ESD) FOR BARRETT’S NEOPLASIA: CLINICAL OUTCOMES AND MID-TERM FOLLOW-UP AT A CANADIAN TERTIARY REFERRAL CENTER

**DOI:** 10.1093/jcag/gwad061.208

**Published:** 2024-02-14

**Authors:** Y Fujiyoshi, K Khalaf, C Natalia, J Mosko, G May, C Teshima

**Affiliations:** Division of Gastroenterology, St. Michael's Hospital, University of Toronto, Toronto, ON, Canada; Division of Gastroenterology, St. Michael's Hospital, University of Toronto, Toronto, ON, Canada; Division of Gastroenterology, St. Michael's Hospital, University of Toronto, Toronto, ON, Canada; Division of Gastroenterology, St. Michael's Hospital, University of Toronto, Toronto, ON, Canada; Division of Gastroenterology, St. Michael's Hospital, University of Toronto, Toronto, ON, Canada; Division of Gastroenterology, St. Michael's Hospital, University of Toronto, Toronto, ON, Canada

## Abstract

**Background:**

Endoscopic submucosal dissection (ESD) is an established technique for resecting early gastrointestinal neoplastic lesions. However, for Barrett’s neoplasia treatment, piecemeal EMR has been the standard approach, leaving the efficacy of ESD for Barrett’s neoplasia unclear.

**Aims:**

To assess the outcomes of Barrett’s neoplasia treated with ESD at a tertiary center in Canada.

**Methods:**

This is a single center, retrospective study. All patients aged over 18 who underwent ESD for Barrett’s neoplasia at St. Michael’s Hospital between December 2017 and August 2023 were included. Primary outcomes were En-bloc, R0, curative resection, and recurrence rates, while secondary outcomes focused on the adverse event rate.

**Results:**

Of the 72 patients studied (mean age 67.7 years; 90% male), 31.9% (23/72) were SSBE and 68.1% (49/72) were LSBE. The mean±SD lesion size was 4.5±3.4 cm, with circumferential occupancy at 53.4±23.9 %. Paris classifications were as follows: 0-Is: 8 (11.1%), 0-IIa: 26 (36.1%), 0-IIb: 9 (12.5%), 0-IIc: 6 (8.3%), 0-IIa+IIc: 13 (18.6%), 0-IIa+Is: 10 (13.9%). The mean operation time stood at 144.4 (75.3) minutes. Adverse events comprised deep mural injury (4.1%) and delayed bleeding (2.8%). Pathology findings showed high-grade dysplasia in 6.9% and adenocarcinoma in 93.1%, further differentiated as: well-differentiated (55%), moderately differentiated (33%), and poorly differentiated (12%). The invasion depth was intramucosal in 76%, SM1 in 6%, and SM2-SM3 in 18%. Lympho-vascular invasion was present in 22%. En-bloc, R0, and curative resection rates were 96% (69/72), 82% (59/72), and 64% (46/72), respectively. Of the curative resection patients, 42 have completed follow-up EGD, with 11.9% (5/42) showing persistent dysplasia at first follow-up. These were treated by either EMR (4 cases) or ESD (1 case), leading to a 100% (42/42) complete remission of dysplasia (CRD) rate with median follow-up time of 127 (68-190) days. After achieving CRD, local recurrence was found in 2.4% (1/42) with a median follow-up time of 289 (136-524) days. This recurrence post-CRD was successfully treated with EMR, regaining CRD.

**Conclusions:**

Our study showcases the Canadian experience of using ESD for Barrett’s neoplasia at a large single tertiary referral center. Given the challenging demarcation lines in Barrett’s neoplasia and the frequent presence of metachronous lesions, even with curative resection, 11.9% showed persistent lesions during the first follow-up. However, these lesions were treatable endoscopically, with 100% eventually achieving CRD. Although there was a 2.4% recurrence rate post-CRD, this too could be managed endoscopically. Overall, the mid-term outcomes of treating Barrett’s neoplasia with ESD are promising.

**Funding Agencies:**

None

